# Hepatitis E Virus Outbreak among Tigray War Refugees from Ethopia, Sudan

**DOI:** 10.3201/eid2902.221495

**Published:** 2023-02

**Authors:** Andrew S. Azman, Etienne Gignoux, Robin Nesbitt, John Rumunu, Rakesh Aggarwal, Iza Ciglenecki

**Affiliations:** Johns Hopkins Bloomberg School of Public Health, Baltimore, Maryland, USA (A.S. Azman);; Médecins Sans Frontières, Geneva, Switzerland (A.S. Azman, I. Ciglenecki);; Epicentre, Paris, France (E. Gignoux, R. Nesbitt);; Ministry of Health South Sudan, Juba, South Sudan (J. Rumunu);; Jawaharlal Institute of Postgraduate Medical Education and Research, Puducherry, India (R. Aggarwal)

**Keywords:** hepatitis E virus, vaccine, displaced populations, viruses, zoonoses, serotype, refugees, Ethiopia, Sudan

**To the Editor:** We read with interest the article by Ahmed et al. on a large hepatitis E virus (HEV) outbreak among refugees from Ethiopia in Sudan, underscoring the challenges in controlling HEV outbreaks (*1*). As part of the rationale for not using HEV vaccine, the authors state that no data on virus genotype were available from cases and “the success of vaccination is dependent on the HEV genotype.” We believe that current evidence contradicts this assertion.

Evidence to date suggests that all major HEV genotypes that infect humans (genotypes 1–4) show cross-protection with a single serotype. Several pieces of data indicate that the only available and licensed vaccine (Hecolin; Wantai BioPharm, https://www.ystwt.cn), which contains recombinant partial capsid protein of HEV genotype 1, offers protection against infection with other genotypes. Studies in rhesus macaques have demonstrated protection by this vaccine against infection with genotypes 1 and 4 (*2*). In a large phase 3 trial of Hecolin, of the 23 persons who had HEV infection (1 in vaccine group and 22 in placebo group), viral genotype was identified in 13 placebo group patients. Of those, 12 were genotype 4 and 1 was genotype 1, providing evidence of protection against genotype 4 infection, a heterologous strain to that in the vaccine (*3*). Furthermore, in vitro data also support cross-protection across HEV genotypes (*4*).

A safe and efficacious vaccine is available and has been recommended for use as an outbreak control tool by the World Health Organization Strategic Advisory Group of Experts on Immunization (*5*), and this recommendation does not refer to virus genotype. Because empirical evidence from in vitro studies, nonhuman primate challenge studies, and a phase 3 clinical trial all point to cross-genotype protection, we believe that the lack of genotyping data during an outbreak should not prevent or delay the use of the HEV vaccine.
